# Reversible Joining Technology for Polyolefins Using Electromagnetic Energy and Homologous Hot-Melt Adhesives Containing Metallic and Ferrite Additives

**DOI:** 10.3390/polym18020228

**Published:** 2026-01-15

**Authors:** Romeo Cristian Ciobanu, Mihaela Aradoaei, George Andrei Ursan, Alina Ruxandra Caramitu, Virgil Marinescu, Rolland Luigi Eva

**Affiliations:** 1 Department of Electrical Measurements and Materials, Gheorghe Asachi Technical University, Bdul. D. Mangeron 71, 700050 Iasi, Romania; mosneagum@yahoo.com (M.A.); andrei_urs@yahoo.com (G.A.U.); darkrollandluigi@yahoo.com (R.L.E.); 2 National Institute for Research and Development in Electrical Engineering (ICPE-CA), 030138 Bucharest, Romania; virgil.marinescu@icpe-ca.ro

**Keywords:** reversible joining technology, hot-melts, polyolefins, inorganic additives, microwave energy

## Abstract

This research examined the development and testing of hot-melt adhesives incorporating metallic (Al and Fe powders averaging 800 nm) and ferrite additives, designed for reversible bonding technology of polyolefins through electromagnetic energy. The experimental models with Al displayed smooth particles that were fairly evenly distributed within the polymer matrix. Experimental models with Fe suggested that Fe nanopowders are more difficult to disperse within the polymer matrix, frequently resulting in agglomeration. For ferrite powder, there were fewer agglomerations noticed, and the dispersion was more uniform compared to similar composites containing Fe particles. Regarding water absorption, the extent of swelling was greater in the composites that included Al. Because of toluene’s affinity for the matrices, the swelling measurements stayed elevated even with reduced exposure times, and the composites with ferrite showed the lowest swelling compared to those with metallic particles. A remarkable evolution of the dielectric loss factor peak shifting towards higher frequencies with rising temperatures was observed, which is particularly important when the materials are exposed to thermal activation through electromagnetic energy. The reversible bonding experiments were performed on polyolefin samples which were connected longitudinally by overlapping at the ends; specialized hot-melts were employed, using electromagnetic energy at 2.45 GHz, with power levels between 140 and 850 × 10^3^ W/kg and an exposure duration of up to 2 min. The feasibility of bonding polyolefins using homologous hot-melts that include metallic/ferrite elements was verified. Composites with both matrices showed that the hot-melts with Al displayed the highest mechanical tensile strength values, but also had a relatively greater elongation. All created hot-melts were suitable for reversible adhesion of similar polyolefins, with the one based on HDPE and Fe considered the most efficient for bonding HDPE, and the one based on PP and Al for PP bonding. When bonding dissimilar polyolefins, it seems that the technique is only effective with hot-melts that include Al. According to the reversible bonding diagrams for specific substrates and hot-melt combinations, and considering the optimization of energy consumption in relation to productivity, the most cost-effective way is to utilize 850 × 10^3^ W/kg power with a maximum exposure time of 1 min.

## 1. Introduction

Hot-melt bonding technologies significantly influence major applications nowadays; their market size was estimated at 10.07 USD Billion in 2024, and is projected to grow from 10.51 USD Billion in 2025 to 16.06 USD Billion by 2035, exhibiting a compound annual growth rate (CAGR) of 4.33% during the forecast period [1]. Hot-melt adhesives are in a molten state and change from liquid at high temperatures to solid at lower temperatures within a specific temperature range. Once the hot melt cools down, it hardens and creates a binding connection between the two selected surfaces [2]. Hot-melt adhesives provide major benefits for high-volume production, including reduced costs due to their easy application, quick bonding, and the use of a single component that does not need additional materials or catalysts for sticking. Conversely, customized hot-melts might be linked to reversible bonding technologies, which are novel ideas associated with the circular economy, primarily driven by the European Community’s recycling efforts across several sectors—including the automotive industry—as per the End-of-Life Vehicles Directive [3]. Reversible bonding may be necessary to detach a temporary structure or assembly that was previously bonded, for reasons such as repair, refurbishment, replacement, or recycling. Streamlined disbanding processes make it easier to recycle materials and components from products and structures bonded with adhesive. Saving frequently on affordable components can be advantageous for lowering assembly or lead time expenses. After the parts have been removed, one can proceed with reconnecting or putting them back together so that the assembled item can be repaired for use.

Hot melt adhesives attach plastic materials through melting, wetting the surface, and quickly solidifying, providing rapid assembly, though adhesion strength can differ significantly. Even if hot-melt bonding technologies are often utilized in various industrial applications to join similar or dissimilar material surfaces in different configurations, effective bonding of polyolefins (mainly high-density polyethylene—HDPE, or polypropylene—PP) may raise technological problems due to their low surface energy. In the case of polyolefins, different hot-melts were tested, but their use necessitated either specific primers or co-polymers [4,5,6,7,8], or preliminary treatment of the surfaces to be bonded, e.g., by Corona, flame, or plasma treatments in order to enhance the surface energy of the polyolefins [9,10,11,12,13].

This paper addresses the development of adapted polyolefin hot-melts to assure the effective bonding of HDPE and PP, starting with the concept that an effective adhesion necessitates that the melted adhesive saturates the plastic surface so materials with similar chemical properties should bond more tightly, e.g., PP based hot-melts would be recommended for bonding PP to PP. This concept describes hot-melt adhesives composed of polyolefin matrices incorporating functionalized metallic and ferrite additives, to assure the chemical compatibility with the substrate and adhesion characteristics, but also to tailor the viscosity of reversible joining technology for polyolefins when using electromagnetic energy. Such polyolefin based hot-melts are supposed to improve the heat resistance and stability of joining compared to the conventional use of Methyl Methacrylate or Ethylene Vinyl Acetate (EVA) hot-melts for polyolefins, especially during their use under specific environmental conditions (improving the endurance at more elevated temperatures or flexibility in cold temperatures) [14]. On the other hand, they are chemically non-reactive and impervious to water (ideal for waterproofing applications), devoid of toxic VOCs, and typically low in scent and ash.

In recent years, technologies connecting electromagnetic energy and hot-melt heating have emerged as a recognized topic in material bonding, closely linked to: (a) the ‘microwave curing’ of a sealant or polymer containing polar groups (even including water) [15,16,17], (b) induction heating of magnetic powders containing hot-melts [18,19,20], and (c) microwave heating of silver or graphene-based sealants utilizing specialized polymers [21,22,23]; all of the above applications fall outside the focus of this paper.

To our knowledge, this research is the first to utilize polyolefin-based hot-melt adhesives with metallic additives for reversible bonding technology of similar polyolefins through microwave energy. It should be noted that all prior publications concerning ‘recyclable hot-melt adhesives with metallic additives, activated by microwave electromagnetic energy’ are authored by the same individuals as in this document. The main applications of this study targets assembly tasks in electronics (e.g., substrate materials, encapsulants, or housing components, for HDPE) [24], and the adherence of internal and external components in lightweight vehicles (e.g., dashboards, door panels, center consoles, etc., for PP) [25]. Essentially, the innovation consists of: (a) direct bonding of polyolefins, which are difficult to adhere otherwise, (b) employing the same matrix in hot-melts as the materials being bonded, (c) utilizing cost-effective materials for producing hot-melts within a circular economy framework, and (d) technological use—mainly in the automotive sector.

## 2. Materials and Methods

### 2.1. Materials and Compounding Technology

Under the concept of a circular economy, recycled matrices of HDPE and PP were used. They were obtained from All Green SRL company (Iasi, Romania), with the manufacturing technology and general features described in [26,27]. The research highlighted that both virgin polyolefins and recycled polyolefins showed nearly identical values concerning thermal and mechanical characteristics, with a maximum variation of 3%. The exception pertains to ash content, which stood at approximately 0.12% for virgin polyolefines and increased to 0.65% for recycled polyolefines, primarily due to residues of CaO, TiO_2_, and SiO_2_. The specific quantity of non-conductive metallic oxides cannot significantly affect the electromagnetic parameters given that the content of conductive or ferritic ingredients was planned to be around 15 times greater, making a study in this area inconsequential.

**Figure 1:**
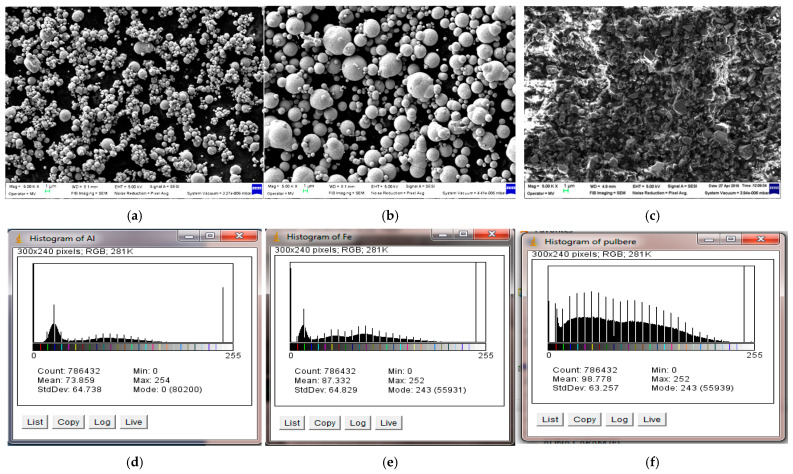
Dimensional analysis of powders: (**a**,**d**) Al; (**b**,**e**) Fe; (**c**,**f**) ferrite.

Composites made with recycled polyolefine powders as the polymer matrix included Al, Fe and ferrite nanofillers. Al and Fe nanofillers originated from the company NANOGRAFI LTD.STI (Ankara Turkey). As regards ferrite powders, SrFe_12_O_19_ were acquired from Mate Co., Ltd. (Wake, Japan) with the trade name HM 1213-PA12 + 85% SrFe_12_O_19_ [27]. The primary features of the metallic powders of Al and Fe, along with their size shape and distribution, are detailed in another source [28]. A brief dimensional analysis of ingredient powders is presented in Figure 1. Al powder exhibited uniform spherical particles, showing reduced diameter variation, with an average size of 765 nm. Fe powder exhibited rough spherical particles with a broad range of diameters, averaging 822 nm, but a consistent presence of larger particles, some even surpassing 2 μm, was evident, with some of them containing smaller particles. Ferritte powder exhibited lamellar particles, characterized by a relatively high dispersion in size, with an average dimension of 792 nm. The existence of larger particles was also observed but their amount was significantly lower in comparison to Fe powder.

A greater variation in particle size (larger for Fe and ferrite particles) was viewed as an advantage, as it was shown to improve adhesive production efficiency, since the wider distribution decreases particle costs by about ten times compared to narrower distributions. Conversely, a wider range of particle sizes resulted in enhanced conductivity and improved dielectric loss characteristics, attributed to a more adequate occupation of the spatial structure in composites, featuring more dispersed waveguides at GHz frequencies and predicting higher values for electromagnetic activation under microwave (MW) conditions of composite hot-melt adhesives [29]. According to the respective simulation results, verified through experiments on real samples, the particle size of 790–800 nm was also discovered to be more beneficial for energy absorption under microwave energy than smaller particles (e.g., of 50 nm), leading to a preference for this size in this study [29,30].

The polymer and nano-conductive powder were mixed for 15 min in a TURBULA T2F cylindrical mixer from Artisan Technology Group (Champaign, IL, USA) to prepare the samples for the experiments, functioning at a rotational speed of 40 rpm. In the end, the composite materials were produced using the Dr. Boy 35A injection machine (Dr. Boy GmbH & Co. K, Neustadt-Fernthal, Germany), which has a screw diameter of 28 mm, an L/D ratio of 18.6 mm, a calculated injection volume of 58.5 cm^3^, a maximum material pressure of 2200 bar, and a minimum effective injection capacity of 500 mm. In each experimental model, 3% of compatibilizing agents was used, which included 1% Poly (ethylene glycol) methacrylate, 1% Ethylene Acrylic Acid Copolymer, and 1% Tegomer^®^ E 525 (Evonik Operations GmbH, Essen, Germany) (wt%).

Table 1 outlines the details of the mixtures. The ideal concentration of 8 wt% metallic powder was determined through extensive simulation using specialized software for S parameters and absorbed microwave energy in polymeric nano-composites containing 5–10 wt% iron and aluminum powder, with two distinct particle sizes, within the frequency range of 0.1–3 GHz [20]. Typically, HDPE and PP melt at higher temperatures (approximately 125–135 °C) than LDPE (around 105–115 °C), which provides HDPE and PP with superior heat performance essential for bonding thermoplastics, in contrast to the findings from [30].

**Figure 2:**
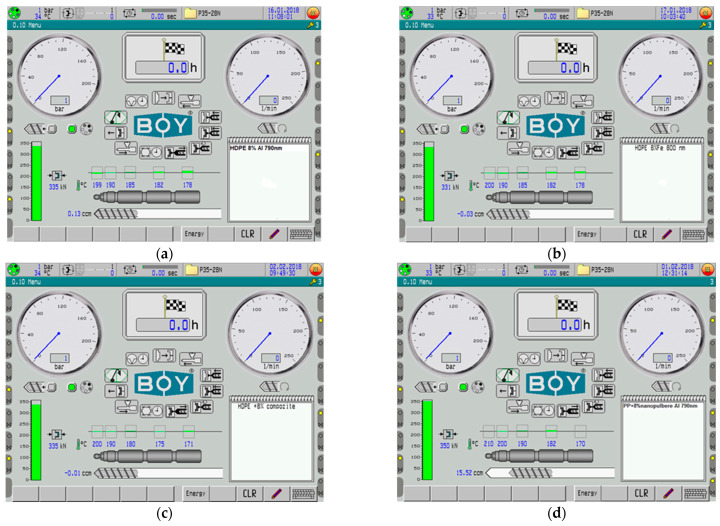

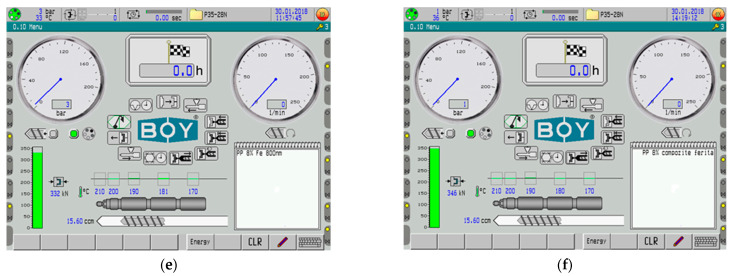
Control monitor of the injection machine, with the temperature settings, for (**a**) M1, (**b**) M2, (**c**) M3, (**d**) M4, (**e**) M5, (**f**) M6.

Figure 2 presents the control monitor of the injection machine, with the temperature settings for the five heating zones, used for the Al- and Fe-containing composites. Temperatures for the Fe-containing composites—as well as for the composites with the PP matrix—had to be fixed somewhat more elevated.

### 2.2. Electromagnetic Bonding Method

**Figure 3:**
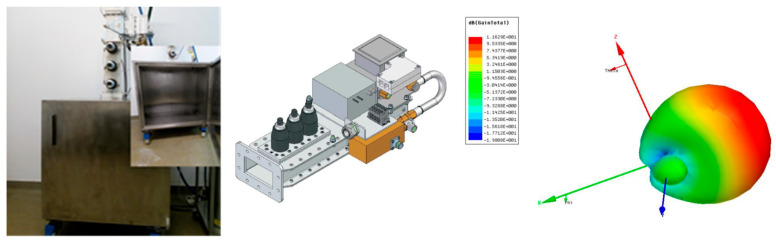
The MW equipment for bonding–disbonding technology and the radiation diagram modeling.

In the case of the LDPE composite hot-melts described in [21], a maximum microwave power of 850 × 10^3^ W/kg was used, at a legally admitted technological frequency of 915 MHz. When utilizing HDPE and PP composite hot-melts featuring metallic inserts, the simulations outlined in [27,29] highlighted through the analysis of S21 transmission parameters that transmission significantly declines as frequency rises within the GHz range, with a saturation effect observed beyond 2.5 GHz. The notable reduction in transmission, linked to minimal reflection, demonstrates that microwave energy is greatly absorbed by HDPE and PP composites containing metallic powder, particularly at frequencies above 2 GHz, thereby supporting their potential application as hot-melt materials. Accordingly, for the electromagnetic bonding technology of HDPE and PP composites with incorporated metallic inserts, the legally admitted technological frequency of 2.45 GHz ± 50 MHz was used. The equipment is presented in Figure 3, using 3 tubes of type MW2009A-260ED and a basic waveguide type WR340 (Muegge GmbH, Reichelsheim, Germany), and providing a maximum power of 1 kW, in line with the maximum power described in [21].

### 2.3. Methods for Characterization and Associated Equipment

(i) Field emission and focused ion beam scanning electron microscopy (SEM) was performed with a Quanta FEG 250 (Thermo Fisher Scientific Inc., Waltham, MA, USA). The analysis method used was LowVac, incorporating water vapor, which protects the samples from damage. The presence of SEM charging was significantly reduced due to the low vacuum in the specimen chamber of the SEM.

(ii) A Netzsch STA PC 409 thermal analyzer (Erich NETZSCH B.V. & Co. Holding KG, Selb, Germany) was employed for thermogravimetric evaluation. The work setting included artificial air at a flow rate of 100 mL/min inside alumina crucibles. The heating program spanned from 35 to 1200 °C, increasing at a rate of 5 °C per minute. The precision of the heat flow measurements was ±0.001 mW.

(iii) The hydrostatic density was assessed with an XS204 Analytical Balance (Mettler-Toledo, Columbus, OH, USA), which has the following specifications: maximum weight limit of 220 g, precision of 0.1 mg, linearity of 0.2 mg, internal calibration, accompanied by a density kit for solids and liquids, and an RS 232 interface (Mettler-Toledo, Columbus, OH, USA). The measurements were conducted at a temperature of 21 °C, consisting of three consecutive repetitions, and the error was assessed. The density was determined as the mean of the three consecutive repeated measurements.

(iv) Shore hardness readings were taken with a standard Shore “D” digital durometer, computed as the mean of 5 measurements, according to ASTM D638-10 [31].

(v) The equipment employed for evaluating the mechanical characteristics was a specific computer-controlled PULL-2000KG Universal Tensile Testing Machine (Lisun Group, Qiantong, Zhejiang, China), featuring a minimum nominal force of 20 kN, facilitating the assessment of tensile strength and elongation, according to ASTM D638-10 [31].

(vi) Microindentation tests were conducted utilizing a compact open platform fitted with a Nano/Micro Indentation Tester and a Micro Scratch Tester, provided by CSM Instruments SA, Peseux, Switzerland. Mechanical tests were performed at room temperature, with five measurements for each sample, and the results’ averages and standard deviations were reported. Vickers hardness (HV) and Young’s modulus (EIT) were obtained through instrumented microindentation tests, following the Oliver&Pharr calculation approach.

(vii) The degree of swelling was evaluated by recording the variation in mass of the samples at designated immersion intervals, utilizing the XS204 Analytical Balance (Mettler Toledo, Columbus, OH, USA).

(viii) The dielectric properties were evaluated with a Broadband Dielectric Spectrometer (Novocontrol GMBH, Montabaur, Germany) that includes an Alpha frequency response analyzer and a Quattro temperature controller, fitted with specialized measurement cells capable of reaching 40 GHz.

(ix) The LFA 447 Nanoflash instrument (Netzsch, Selb, Germany) was employed to assess thermal conductivity, adhering to the ASTM E-1461:2007 standard [32], by using the “flash” technique. A powerful xenon lamp acted as the radiation energy source, and the time of exposure on the sample’s front surface was 0.18 ms. The samples were analyzed three times at each temperature. The increase in temperature on the other side of the sample was measured with an InSb type infrared (IR) detector.

(x) The Raman spectra were collected with a Fluorolog-3 spectrophotometer, model FL3-2.2.1, manufactured by Horiba Jobin Yvon (Palaiseau, France). The spectra were captured in backscattering geometry, employing an excitation wavelength of 1064 nm.

(xi) The analysis to measure the surface topography of the samples was conducted using atomic force microscopy, employing the Dimension Edge instrument from Bruker (Billerica, MA, USA). The roughness was calculated as the average of 3 measurements taken in the central area of the same sample with a focal length of 60 μm.

## 3. Results and Discussion

### 3.1. Raman Spectra Analysis

**Figure 4:**
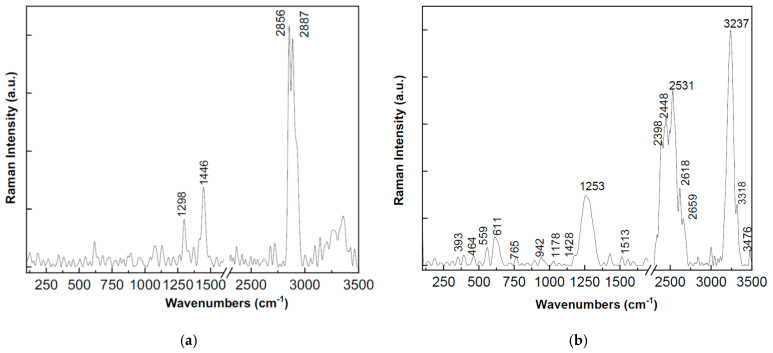

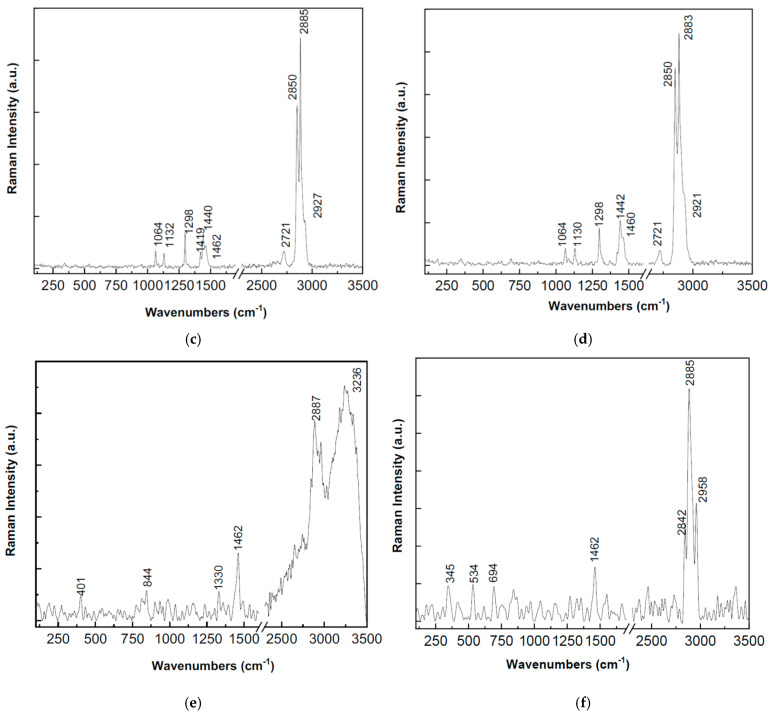
Raman spectra recorded for the analyzed samples: (**a**) HDPE with Al; (**b**) HDPE with Fe; (**c**) HDPE with ferrite; (**d**) PP with Al; (**e**) PP with Fe; (**f**) PP with ferrite.

The analysis of the Raman spectra presented in Figure 4 indicated the following: In the case of the samples with HDPE matrix: Figure 4a, Al presents four intense lines at 1298, 1446, 2856, and 2887 cm^−1^; Figure 4b, Fe presents three intense lines with maxima at ca. 1253, 2398, 2448, 2531 and 3237 cm^−1^, accompanied by other low intensity Raman lines at ca. 559 and 611 cm^−1^; Figure 4c, the ferrite presence is identified by a spectrum that is dominated by the lines at 2850, 2885–2927 cm^−1^, accompanied by four lines in the spectral range 1000–1600 cm^−1^, having maxima at 1064, 1132, 1298 and 1440 cm^−1^. The Raman lines reported for the HDPE + Al sample at about 1067, 1132, 1299 and 1445 cm^−1^ are specific to HDPE, these being attributed to the first two vibrational modes of stretching the C-C bond in the trans CH_2_ groups, the torsional vibration mode in the CH_2_ group and the CH_2_ bond vibration mode, respectively. The Raman spectrum of the HDPE + Fe sample is slightly different from the one mentioned above, in this case the Raman spectrum lines belonging to HDPE were noted to be located at 1178 and 1428 cm^−1^ and were attributed to the tilting vibrational mode of the CH^2^ bond in the crystal structure and the bond vibration mode of the CH^2^ group.

In the case of the samples with PP matrix: Figure 4d, Al shows the following main Raman lines 1298, 1442, 2850 and 2883 cm^−1^; Figure 4e, Fe shows two intense bands at ca. 2887 and 3236 cm^−1^ which are accompanied by four other lines of lower intensity at ca. 401, 844, 1330 and 1462 cm^−1^; Figure 4f, presents the ferrite in addition to the Raman lines at 1462, 2842 and 2885–2958 cm^−1^, three low intensity lines in the spectral range 250–750 cm^−1^ with maxima at ca. 345, 534 and 694 cm^−1^. Raman spectra were reported for PP matrix and the lines 844, 1330, 1460 and 2887 cm^−1^ were assigned to the vibrational modes of syndiotactic polypropylene, the hexagon unit cell of isotactic polypropylene, the symmetric deformation vibrational mode of the methyl group of isotactic polypropylene, and the symmetric stretching vibrational mode of the C-H bond of the methyl group of isotactic polypropylene, respectively.

### 3.2. Afm Analysis

**Figure 5:**
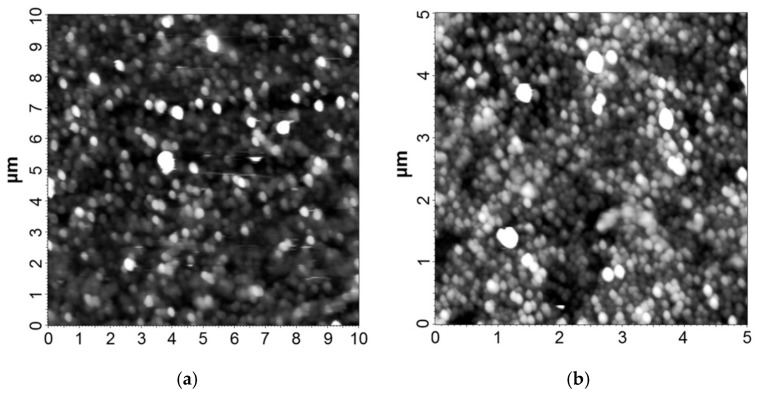
AFM microscopy images for (**a**) HDPE + 8% Al 800 nm, (**b**) HDPE + 8% Fe 800 nm.

For composite samples containing polyolefin matrices, and given the lower precision of the injection technology, assessing the surface roughness was challenging; however, roughness values between 75 and 450 nm were achieved, indicating the limitations of atomic force microscopy analysis. In Figure 5, AFM microscopy images for the samples of HDPE with Al and PP with Fe are presented, for which the analysis results are considered most relevant, namely those with roughness below 100 nm.

### 3.3. Sem Analysis

**Figure 6:**
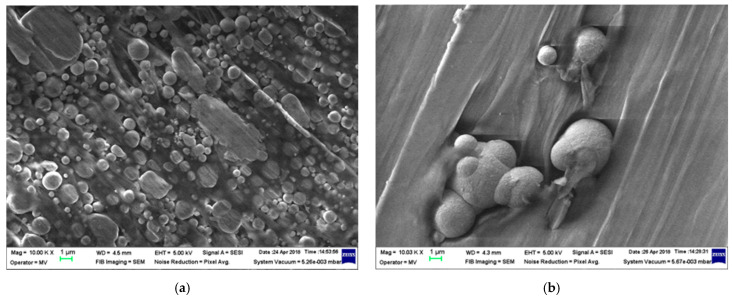

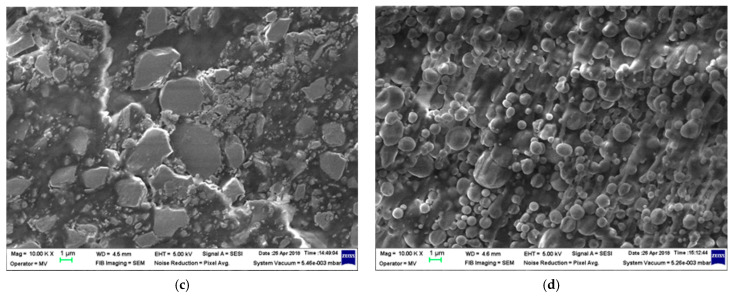

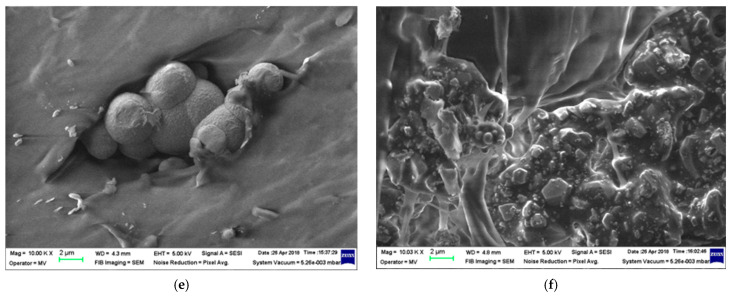
Micrographs for (**a**) M1; (**b**) M2; (**c**) M3; (**d**) M4; (**e**) M5 and (**f**) M6.

In Figure 6, the micrographs made for the samples M1–M6 are presented.

It was noted that the experimental models containing Al (M1, Figure 6a, respectively, M4, Figure 6d) presented smooth particles, with a relatively uniform distribution in polymer mass. Experimental models containing Fe outlined that the Fe nanopowders (presenting rough spherical particles) are more difficult to be dispersed in the polymer mass. As illustrated in Figure 1b, the presence of dimensionless particles with greater diameters accounts for the adhesion of other particles to these larger ones and could lead to local agglomerations. However, these agglomerates are not the norm; generally, other particles are evenly distributed throughout the polymer matrix. In the case of ferrite powder, it was found that the most homogeneous was M3 compared to M6, and in the case of the HDPE matrix, the dispersion more uniform. The spatial structure of composites with ferrite is significantly more uniform compared to similar composites containing Fe particles.

### 3.4. Density Analysis

The results of the hydrostatic density analysis are presented in Table 2.

The samples based on the HDPE matrix show lower densities compared to the samples based on the PP matrix, for all types of powders. For both types of polymeric matrices, the highest density was achieved by composites with Fe powder, followed by ferrite powder.

### 3.5. Mechanical Tests

The results of the Shore hardness and mechanical tests are presented in Table 3 and Table 4, according to ASTM D638-10 [31].

Regarding Shore hardness, the samples based on the HDPE matrix show lower values compared to the samples based on the PP matrix, for all types of powders. The highest values are achieved by composites with ferrite powder, and the lowest for the composites with Al powder, for both types of polymeric matrices.

Regarding the mechanical test results, it was observed that for resistance and Young’s modulus, the composites using a PP matrix showed higher values for all powder types, whereas the elongation results are contrary. The lowest mechanical resistance and highest values for the flow resistance and elongation were achieved by the composites with Al powder, for both types of polymeric matrices.

### 3.6. Microindentation Tests

The microindentation tests results for the composite samples are presented in Table 5.

The mechanical properties, particularly indented hardness HIT and Vickers hardness HV, are somewhat greater for composites with Fe and with PP matrix, irrespective of the metal type. M5 achieved the highest values. The most reduced modulus of elasticity EIT was discovered for Fe containing samples. The elastic indentation work and reversible elastic deformation work exhibit greater values for samples with Al—the highest values were reached by M3. Concerning the mechanical plastic deformation of indentation Wplastic, somewhat higher values were observed for composites that included Fe powder, such as M2. The microindentation analysis revealed clear differences in the surface structure of the samples, associated with the nature of additives and their effects on the interaction with the matrix during compounding, in line with the findings related to SEM analysis.

### 3.7. Thermogravimetric Measurements

**Figure 7:**
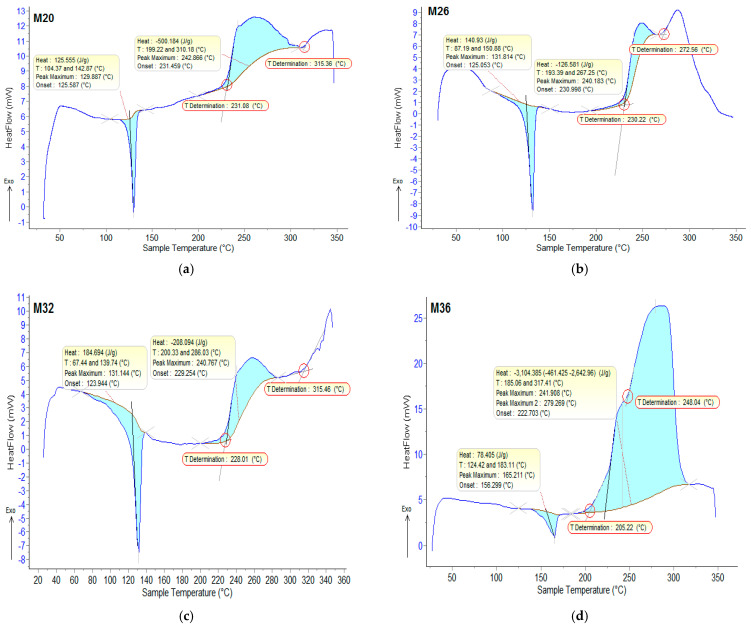

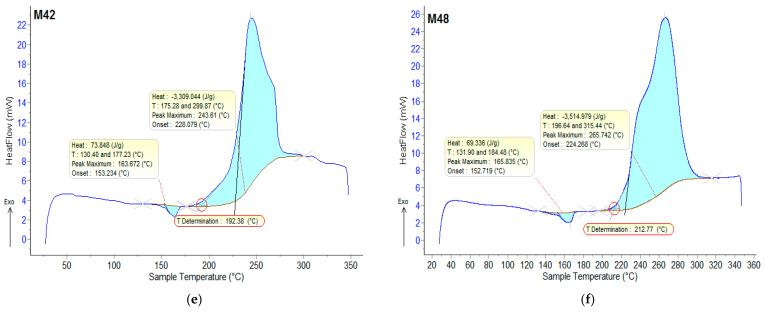
DSC-recorded curves for the composite samples: (**a**) M1; (**b**) M2; (**c**) M3; (**d**) M4; (**e**) M5 and (**f**) M6.

In Figure 7, the DSC curves for the analyzed composites are presented, along with the analysis of the thermogravimetric data in Table 6.

Susceptibility to oxidation depends on the chemical structure of the polymer, the degree of crystallinity of the polymer, as well as the susceptibility to oxidation of the metal/ferrite particles used as fillers. In these types of composite materials, from the statistical interpretation of the results obtained, it was found that the melting temperatures are very similar for the composites with the same matrix, varying in the range of 129.9–131.8 °C for HDPE and in the range of 163.7–165.8 °C for PP. The temperatures for the start of the first oxidation process vary in the range of 192.4–231 °C, being higher for HDPE. ΔH_t_ presented much higher values in the case of the composites with HDPE matrix. The temperatures of the start of the second oxidation process (OOT_2_) varied in the range of 248–315.5 °C for the samples M1–M4, but were missing for the samples M5 and M6. Further heating the composites led to thermo-oxidative processes until complete destruction of the polymer matrices.

### 3.8. Thermal Conductivity Tests

The results of the thermal conductivity tests are outlined in Table 7, according to ASTM E-1461:2007 [32]. Generally, the composites using a PP matrix showed higher values for all powder types. The composites using a PP matrix showed higher values for all powder types. As expected, the composites with metallic powder presented higher values compared to ferrite containing samples, for both matrix types, the best results were achieved by the composites with Al.

### 3.9. Results Obtained for the Degree of Swelling in Water and Solvent

The procedure was carried out according to SR EN ISO 175/2011 [33], and the results are presented in Table 8 and Table 9.

Incorporating metallic and ferrite particles into polymer matrices can generate micro-voids during melting, influencing the surface roughness of the samples and modifying liquid adhesion and penetration ability. Regarding water absorption, as shown in Table 8, it was noted that the swelling remained minimal until 240 h of immersion, after which it rose rapidly, reaching a saturation level near 500 h, which was nearly double the value recorded at 240 h. The degree of swelling was elevated in the composites containing Al, due to the increased occurrence of micro-voids in the materials as a result of the particle dispersion mentioned in the SEM analysis.

Regarding the swelling levels associated with toluene, as indicated in Table 9, it was noted that due to toluene’s attraction to the matrix, the values were high even with brief immersion durations, and the rise in values with longer immersion times is consistent. The general trend has shifted, with composites containing ferrite exhibiting the minimal degree of swelling in comparison to those with metallic particles. Regarding the solvent, there was no saturation observed, and the swelling degree was fairly consistent across all composites containing metallic particles, indicating that toluene uptake is not significantly influenced by the quantity or structure of micro-voids.

### 3.10. Dielectric Tests

The dielectric tests are essential for the application of hot-melts when thermal activation by electromagnetic energy is supposed. Extensive tests upon the dielectric parameters—mainly dielectric permittivity, dielectric loss factor (Tan Delta), and conductivity—were performed in the frequency range from 100 MHz to 3 GHz, to cover the domain of interest for the use of microwave energy, as preliminary theoretically simulated in [27]. The tests were performed at two different temperatures, at room temperature of 20 °C and also at 100 °C, to better understand the dielectric behavior of composites during the melting process. As regards the dielectric permittivity, a slight increase in the characteristic was noticed for all samples, starting from 1 GHz, followed by a saturation after 2 GHz, for both analyzed temperatures. The difference in values from the room temperature of 20 °C and, respectively, at 100 °C was less than 3% in all circumstances. The highest values were obtained for Al inserts and for PP matrix, but the differences were minor compared to the other samples. The same conclusions were obtained as regards the evolution of conductivity, and it was concluded that their impact upon the melting process is minor, compared to the dielectric loss factor.

**Figure 8:**
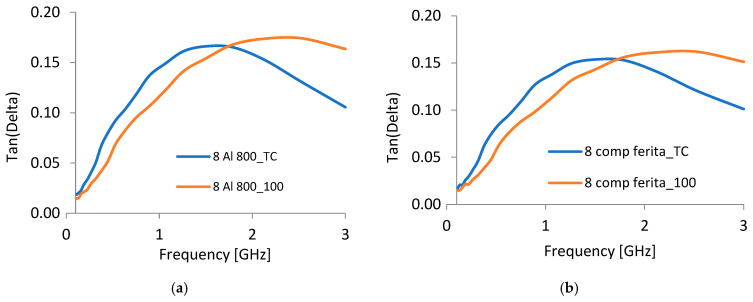
Dielectric loss factor evolution for: (**a**) M1 and (**b**) M6.

In Figure 8, an example of the evolution of the dielectric loss factor is outlined for both analyzed temperatures (room temperature of 20 °C—TC as well as at 100 °C). It should be noted that this kind of remarkable evolution is similar for both matrices and all types of inserted particles, even if the maximum values for Al containing composites are slightly higher compared to the composites with the other inserts, for both matrices types. A possible explanation could be related to the elevated mobility of charge carriers in waveguides and enhanced polarization activities caused by increased dipole mobility near progressively heated particles, along with the development of an additional ionic conduction, specific for the GHz frequency domain.

The phenomenon of the transition of the maximum of dielectric loss factor characteristic towards higher frequencies at elevated temperatures is relevant for the application of hot-melts when thermal activation by electromagnetic energy is performed, facilitating the melting procedure in an efficient way.

According to the discussion in [29], for HDPE and PP composites with incorporated metallic powder, microwave energy is significantly absorbed by the materials, mainly in the case of frequencies exceeding 2 GHz. Thus, at frequencies greater than 2 GHz, the peculiar behavior of the dielectric loss factor near the melting point offers benefits regarding the energy efficiency of bonding technology. In this case, utilizing the 2.45 GHz frequency for bonding maintains a nearly constant energy transfer at its peak during the heating of hot-melts, demonstrating a greater efficiency when compared to similar hot-melts formulated with the LDPE matrix, for which a frequency of 915 MHz was recommended [30].

### 3.11. Thermal Endurance Tests

**Figure 9:**
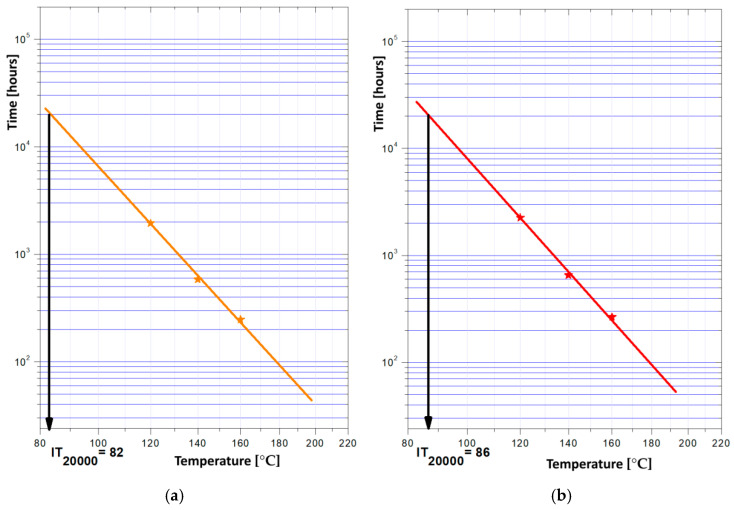

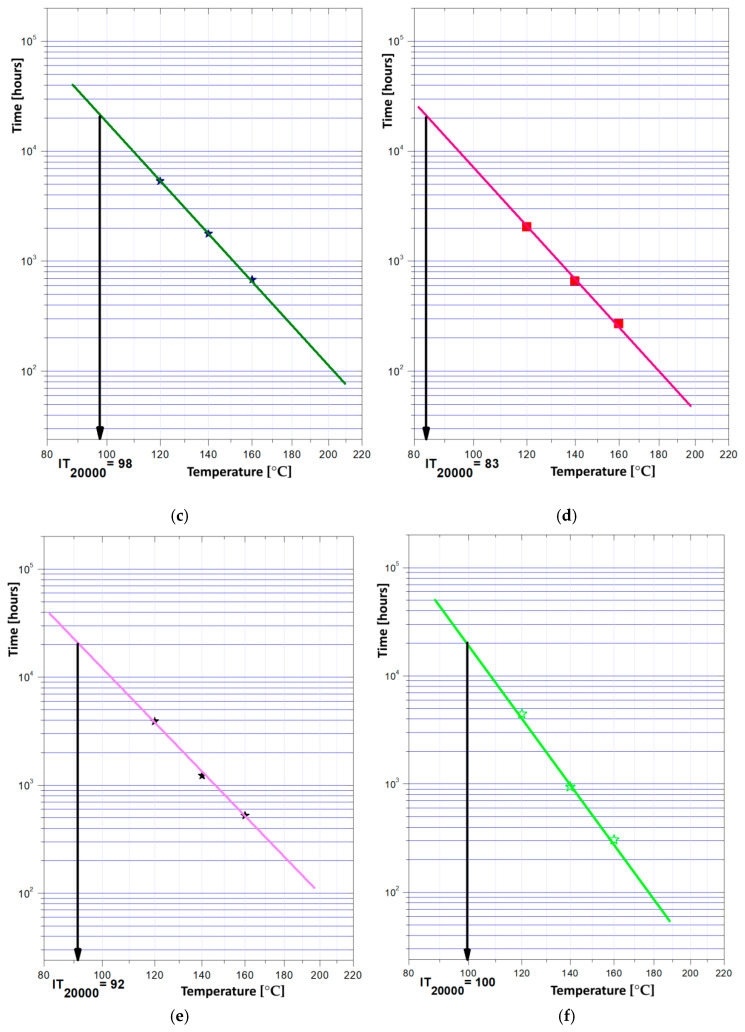
Arrhenius diagrams with the index point at 20,000 h of operation for: (**a**) M1; (**b**) M2; (**c**) M3; (**d**) M4; (**e**) M5 and (**f**) M6.

For these composite materials, an accelerated aging test was applied, as presented in Table 10, with the minimum exposure temperature chosen to be 120 °C, and the maximum 160 °C. To interpret the data obtained from these tests, the Arrhenius equation was applied, which establishes the dependence of the chemical degradation reaction coefficient on temperature, adapted to the approximation of the relationship between the material’s lifespan and temperature. The degradation criterion chosen for these materials was mass loss, by considering that the end of life of the materials would be a mass loss of 10% (equivalent of the temperature index at 20,000 h of operation). The Arrhenius diagrams are presented in Figure 9, with the index at 20,000 h of operation for each sample; the values are summarized in Table 11.

The highest thermal stability presents the composites with ferrite, and in all, higher values present the composites with PP matrix. The lowest value is offered by M1, of 82 °C, but clearly exceeds the imposed maximum value of 70 °C for service exposure in the targeted applications of assembly tasks in electronics and adhering internal and external components in lightweight vehicles. It should be noted that the values obtained above pertain to the thermal exposure of materials in accordance with the electrical engineering standard [34], specifically regarding the insulation class; in our instance, they were equated to the insulation class A for automotive electronics and components, such as housings, etc. (105 °C usage limit, 70 °C exposure to assess lifespan—in our case surpassing 20,000 h for all samples, with a maximum ambient temperature of 40 °C)**.**

## 4. Bonding Technology Concept and Evaluation

Microwave heating is one of the fastest and most efficient methods for heating materials, differing from conventional heat transfer at a fundamental level. While conventional heating works by transferring heat from the surface of an object to its center, microwaves deliver heat uniformly and simultaneously throughout the entire volume of a material, starting from the center. This difference can provide unique advantages for microwave effective reversible bonding of polyolefins-based components in many industrial applications. The application of homologous hot-melt adhesives with metallic additives offers an extra advantage, as the electromagnetic energy applied is focused on the hot melt mass, causing its melting and ultimately the bonding process, while not impacting the components being bonded.

**Figure 10:**
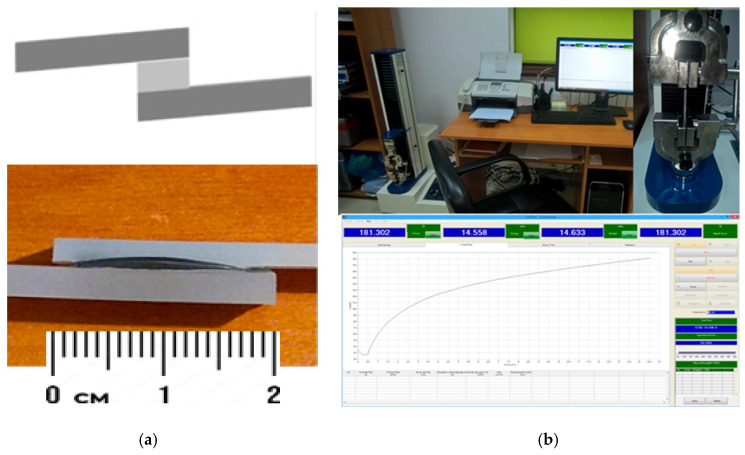
Bonding concept and test: (**a**) longitudinal joint and (**b**) mechanical strength equipment.

The tests were made on polyolefin samples, longitudinally joint by overlapping at the ends (materials thickness—3 mm, bonded area—2 × 1 cm^2^), with the addition of tailored hot-melts (hot-melt thickness—0.8 mm), Figure 10a. The testing of the joint mechanical parameters was performed with the equipment described at 2.3, Figure 10b. The applied electromagnetic energy varied in the range of 140–850 × 10^3^ W/kg. The exposure time for each bonding–disbonding technology varied up to 2 min, the most reasonable time in terms of efficiency.

**Figure 11:**
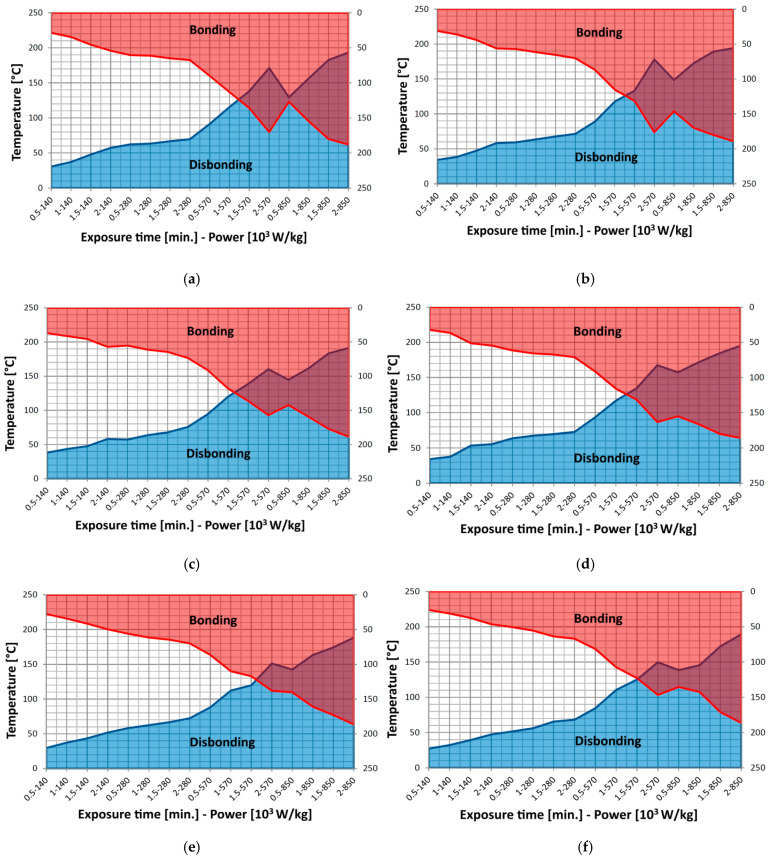

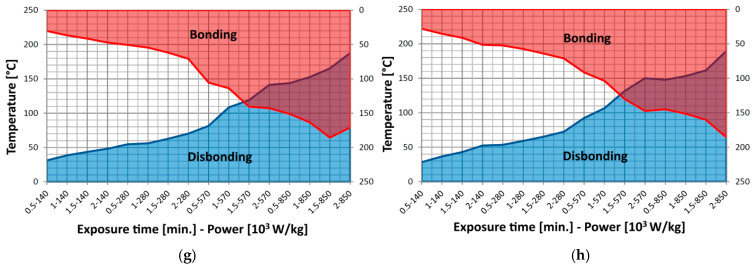
Reversible bonding diagrams for: (**a**) SL1; (**b**) SL2; (**c**) SL3; (**d**) SL4; (**e**) SL5; (**f**) SL6; (**g**) SL7; (**h**) SL8.

A synthesis of the bonding technology concept and related test results is presented in Table 12. The reversible bonding diagrams for the type of substrates and hot-melt combinations (described in Table 12) are presented in Figure 11, demonstrating the validity of the concept of disbonding through the use of identical microwave exposure.

Analyzing the results in Table 12, the feasibility of bonding polyolefins by using homolog hot-melts with metallic/ferrite ingredients is validated. For both matrices, the hot-melts including Al presented the highest values of mechanical tensile strength, but also a relatively higher elongation. The hot-melts with Fe provided inferior performances. It was also demonstrated that bonding of substrate of HDPE with PP is possible with both types of hot-melts, and the results are reasonable in terms of mechanical strength, even if the hot-melt based on PP resin brings slightly better results.

Concerning the outcomes of the reversible bonding technology shown in Figure 11, it should be emphasized that a microwave source power inferior to 570 × 10^3^ W/kg is impractical, as the required exposure time is insufficient to guarantee the melting of the hot-melts. The optimal bonding–disbonding technological region is highlighted by the intersection of the two technological fields, namely the shaded area in Figure 11. In principle, the largest temperature domain at a certain power and time of exposure would represent the most feasible solution, and consequently this concept should nevertheless indicate the domain with the highest power applied and largest exposure time (e.g., about 130 °C at 850 × 10^3^ W/kg power and 2 min) in all circumstances. It is obvious that this concept needs to be refined and linked to energy efficiency (reduced power usage) and productivity (minimal exposure duration) to guarantee technological effectiveness. It is presumed that a minimally acceptable temperature range to ensure a feasible reversible bonding process would be approximately 50 °C in all situations. In all technological trials, it was noticed that a minimal duration of 1.5 min is needed at 570 × 10^3^ W/kg power, but the temperature domain is restricted to one point. In the technological cases of SL1-SL6 and SL8, a first reasonable domain is reached for 2 min at 570 × 10^3^ W/kg power. This domain is not reached in the case of SL7, where it occurs only at an exposure of 1 min at 850 × 10^3^ W/kg power. The second domain is reached at a 850 × 10^3^ W/kg power as follows: 1 min for SL1, SL3, SL5 and SL8; 0.5 min in the case of SL2 and SL4, and 1.5 min in the case of SL6.

When optimizing the energy consumption relative to productivity, it is essential to highlight the minimum energy value for the shortest exposure time. Finally, the ideal parameters for the reversible bonding technology, pertaining to the case examined in this study, are shown in Table 13. All manufactured hot-melts were suitable for the reversible bonding process of similar polyolefins, but as the most efficient were appointed M2 for bonding HDPE, and M4 for PP bonding. In the case of bonding dissimilar polyolefins, it seems that the process is efficient only by using hot-melts containing Al, but the mechanical parameters are somewhat reduced.

The reversible bonding method also allows for the complete recovery of dismantled components and the recycling of hot-melts, which can be reprocessed and utilized with the same bonding technology, a concept in line with the circular economy requirements.

## 5. Conclusions

This study presents the manufacturing and testing of hot-melt adhesives with metallic (Al and Fe powders with an average dimension of 800 nm) and ferrite additives, aimed at reversible bonding technology for polyolefins utilizing electromagnetic energy. The experimental models containing Al exhibited smooth particles with a fairly uniform distribution throughout the polymer mass. Experimental models involving Fe indicated that the Fe nanopowders are more challenging to disperse in the polymer matrix, often leading to agglomeration. For ferrite powder, it was observed to be fewer agglomerations and the dispersion is more consistent, notably more consistent than that of comparable composites with Fe particles.

Integrating metallic and ferrite particles into polymer matrices can create micro-voids during melting, affecting the surface roughness of the samples and altering liquid adhesion and penetration capacity. Regarding water absorption, the level of swelling was greater in the composites that included Al. Because of toluene’s affinity for the matrices, the swelling values stayed elevated even with reduced exposure times, and the composites with ferrite showed the lowest swelling compared to those featuring metallic particles.

A remarkable phenomenon of shifting of the peak of the dielectric loss factor towards higher frequencies at increased temperatures was noticed, which was very significant for the use of hot-melts when subjected to thermal activation via electromagnetic energy. The hot-melts based on HDPE and PP, containing metallic and ferrite powder, absorb microwave energy considerably, especially at frequencies above 2 GHz. Accordingly, the reversible bonding tests were conducted on polyolefin samples, longitudinally joined by overlapping at the ends, incorporating custom hot-melts, utilizing electromagnetic energy at 2.45 GHz, with the power in the range of 140–850 × 10^3^ W/kg and with an exposure duration up to 2 min. The practicality of joining polyolefins through the application of homolog hot-melts containing metallic/ferrite components was confirmed. For both matrices, the hot-melts containing Al exhibited the greatest mechanical tensile strength values, but also had a comparatively higher elongation.

All the produced hot-melts were appropriate for the reversible bonding of similar polyolefins, with M2 deemed the most effective for bonding HDPE and M4 for bonding PP. When bonding different polyolefins, it appears that the method is effective solely with hot-melts containing Al, although the mechanical properties are slightly diminished. Based on the reversible bonding diagrams for the type of substrates and hot-melt combinations—and taking into account the optimization of the energy consumption relative to productivity, the minimum energy value for the shortest exposure time was highlighted, leading to the conclusion that it is more economical to use 850 × 10^3^ W/kg power at a maximum 1 min exposure time.

The reversible bonding technique enables the full retrieval of disassembled parts and the recycling of hot-melts, which can be reprocessed and used with the identical bonding technology.

## Figures and Tables

**Table 1:** Mixtures’ descriptions (addition in wt%).

Sample Code	Formulation
M1	rHDPE + 8% Al + 3% Additives
M2	rHDPE + 8% Fe + 3% Additives
M3	rHDPE + 8% Ferrite + 3% Additives
M4	rPP + 8% Al + 3% Additives
M5	rPP + 8% Fe + 3% Additives
M6	rPP + 8% Ferrite + 3% Additives

**Table 2:** Results of the density analysis.

Sample	Density (g/cm^3^)
M1	0.924
M2	0.953
M3	0.941
M4	0.959
M5	0.976
M6	0.967

**Table 3:** Results of the Shore hardness.

Sample	Average Shore Hardness A (HS)
M1	55
M2	59
M3	60
M4	59
M5	63
M6	67

**Table 4:** Results of the mechanical tests.

Sample	Mechanical Resistance Rm [MPa]	Flow Resistance Rp [MPa]	Elongation A [%]	Young’s Modulus [GPa]
M1	14.47	0.37	108	0.39
M2	16.36	0.24	98	0.53
M3	17.71	0.28	94	0.56
M4	14.88	0.41	102	0.44
M5	16.98	0.32	88	0.61
M6	18.22	0.36	79	0.73

**Table 5:** Microindentation tests results.

Sample	H_IT_ (MPa)	HV	E_IT_ (GPa)	S (N/µm)	h_max_ (µm)	W_elastic_ (µJ)	W_plastic_ (µJ)	W_total_ (µJ)	η_IT_ (%)
M1	54.1 ± 1.6	5.1 ± 0.2	1.2 ± 0.14	0.22 ± 0.03	30.9 ± 0.2	3.3 ± 0.1	8.2 ± 0.2	11.5 ± 0.2	28.5 ± 0.6
M2	78.0 ± 3.5	7.4 ± 0.3	0.9 ± 0.01	0.14 ± 0.01	27.8 ± 0.2	1.1 ± 0.2	9.2 ± 0.5	10.3 ± 0.4	10.9 ± 2.0
M3	64.6 ± 9.7	6.1 ± 0.9	1.1 ± 0.13	0.18 ± 0.03	29.7 ± 0.4	3.6 ± 0.3	8.0 ± 0.6	11.6 ± 0.9	30.7 ± 0.2
M4	97.4 ± 19.8	9.2 ± 1.9	1.4 ± 0.16	0.16 ± 0.01	25.5 ± 2.3	3.4 ± 0.1	5.5 ± 0.3	8.9 ± 0.4	38.2 ± 0.1
M5	104.3 ± 14.1	9.8 ± 1.3	1.3 ± 0.07	0.18 ± 0.02	23.9 ± 0.9	3.2 ± 0.1	5.7 ± 0.1	8.7 ± 0.1	37.0 ± 0.1
M6	103.8 ± 21	9.8 ± 2	1.5 ± 0.04	0.21 ± 0.02	23.6 ± 1.8	2.5 ± 0.7	6.0 ± 0.4	8.5 ± 1.0	28.9 ± 4.3

**Table 6:** Analysis of the thermogravimetric data.

Sample	T_t_ (°C)	ΔH_t_ (J/g)	χ_cr_ (%)	OOT_1_ (°C)	OOT_2_ (°C)
M1	129.9	125.5	46.5	231	315.4
M2	131.8	140.9	52.2	230.2	272.5
M3	131.1	184.7	68.5	228	315.5
M4	165.2	78.4	41.1	205.2	248
M5	163.7	73.8	38.7	192.4	-
M6	165.8	69.3	36.4	212.8	-

**Table 7:** Thermal conductivity results.

Sample	Thermal Conductivity (W/(m*K)
M1	0.252
M2	0.241
M3	0.233
M4	0.304
M5	0.264
M6	0.251

**Table 8:** Degree of swelling in water, at different immersion times [%].

Sample	Q Water 72 h	Q Water 168 h	Q Water 240 h	Q Water 336 h	Q Water 408 h	Q Water 504 h	Q Water 576 h
M1	1.7866	3.9336	5.8172	7.4451	8.8801	10.6814	10.6834
M2	1.0067	1.9934	2.9605	3.9088	4.8387	6.2498	6.2518
M3	1.3232	2.4444	3.5423	4.3555	5.5657	6.6050	6.6070
M4	1.8759	3.6828	5.4243	7.1040	8.7250	9.9440	9.9440
M5	0.9297	1.8423	2.7382	3.6179	4.4819	5.6663	5.6663
M6	1.2288	2.1308	3.0164	3.8862	4.7405	6.2583	6.2583

**Table 9:** Degree of swelling in toluene at different immersion times [%].

Sample	Q _toluene_ 72 h	Q _toluene_ 168 h	Q _toluene_ 240 h	Q _toluene_ 336 h	Q _toluene_ 408 h	Q _toluene_ 504 h	Q _toluene_ 576 h
M1	8.2747	8.7031	9.8129	9.6596	10.7580	11.8056	11.8081
M2	9.4533	9.3585	9.8262	10.7390	11.3286	11.1341	11.1366
M3	9.1263	9.0407	9.5821	10.0196	10.7968	10.9435	10.9460
M4	8.5626	8.7265	10.1008	9.6719	11.0459	11.8179	11.8204
M5	9.7412	9.3819	10.1141	10.7514	11.6165	11.1464	11.1489
M6	9.4142	9.0641	9.8700	10.0319	11.0847	10.9559	10.9584

**Table 10:** Accelerated aging program.

Temperature [°C]	Exposure Time (Hours)
120	120
140	72
160	24

**Table 11:** Values of IT 20,000 [°C].

Sample	IT 20,000 [°C]
M1	82
M2	86
M3	98
M4	83
M5	92
M6	100

**Table 12:** Bonding details and test results.

Bonded Samples Code	Type of Substrates and Hot-Melt Combination	Mechanical Tensile Strength of the Joint [MPa]	Elongation [%]
SL1	Substrate of HDPE-M1	7.28	31
SL2	Substrate of HDPE-M2	6.84	28
SL3	Substrate of HDPE-M3	7.11	33
SL4	Substrate of PP-M4	7.12	25
SL5	Substrate of PP-M5	6.75	21
SL6	Substrate of PP-M6	6.78	24
SL7	Substrate of HDPE with PP-M1	6.83	32
SL8	Substrate of HDPE with PP-M4	6.93	27

**Table 13:** Optimal parameters for the reversible bonding technology.

Bonded Samples Code	Type of Substrates and Hot-Melt Combination	Applied Microwave Power [×10^3^ W/kg]	Exposure Time [min]
SL1	Substrate of HDPE-M1	850	1
SL2	Substrate of HDPE-M2	850	0.5
SL3	Substrate of HDPE-M3	850	1
SL4	Substrate of PP-M4	850	0.5
SL5	Substrate of PP-M5	850	1
SL6	Substrate of PP-M6	570	1.5
SL7	Substrate of HDPE with PP-M1	850	1
SL8	Substrate of HDPE with PP-M4	850	1

## Data Availability

The original contributions presented in this study are included in the article. Further inquiries can be directed to the corresponding authors.
